# Anti-CENP-B polarity divides SLE: divergent clinical-immune phenotypes and distinct treatment responses

**DOI:** 10.3389/fimmu.2026.1762899

**Published:** 2026-01-22

**Authors:** Xiaoli Liu, Zhefeng Xiao, Yuxing Yao, Youhua Yuan, Xia Zhang, Jianfeng Li, Xiuzhi Zhang, Xiaohui Tian, Lemei An

**Affiliations:** 1 Department of Clinical Laboratory, Henan Provincial People’s Hospital, People’s Hospital of Zhengzhou University, Zhengzhou, Henan, China; 2 Department of Pathology, NHC Key Laboratory of Cancer Proteomics, National Clinical Research Center for Geriatric Disorders, Xiangya Hospital, Central South University, Changsha, China; 3 College of Letters and Science, University of California, Berkeley, Berkeley, CA, United States; 4 Department of Pathology, Henan Medical College, Zhengzhou, China; 5 Department of Rheumatology and Immunology, Henan Provincial People’s Hospital, People’s Hospital of Zhengzhou University, Zhengzhou, China

**Keywords:** anti-CENP, immune cell, limited cutaneous systemic sclerosis, multivariate regression, systemic lupus erythematosus (SLE), therapeutic response

## Abstract

**Background:**

Anti-CENP-B antibodies (anti-CENP-B), directed against centromere protein B, are a serological hallmark of limited cutaneous systemic sclerosis (lcSSc) but are only occasionally encountered in systemic lupus erythematosus (SLE). When detected in SLE they may create diagnostic ambiguity. Since autoantibody-defined SLE subsets exhibit distinct phenotypes, delineating the clinical and immunological features of anti-CENP-B-positive disease is essential for precise management.

**Methods:**

We retrospectively collected demographic, clinical, laboratory and therapeutic data from 310 SLE patients including 73 anti-CENP-B-positive patients and 237 anti-CENP-B-negative patients. Inter-group differences, correlations, and multivariable logistic regression were performed.

**Results:**

Compared with the anti-CENP-B-negative patients, the anti-CENP-B-positive patients were older, less frequently had lupus nephritis (LN), but more often exhibited Raynaud’s phenomenon, cardiac, or pleuropulmonary involvement. Serologically, they displayed lower anti-dsDNA, anti-nucleosome and anti-histone antibody levels, reduced C4, yet higher IgA, IgM, and IgG concentrations and expanded CD19^+^ B-cell numbers; accordingly, SLEDAI-2K scores and 24-h urinary protein (24h-UTP) were lower. C4 inversely correlated with disease activity indices and IgG in the positive group, whereas in the negative group it also correlated with B-cell counts and IgM levels. Multivariate logistic regression identified age, Raynaud’s phenomenon, CD19^+^ B-cell count, and IgG level as factors independently associated with anti-CENP-B positivity. Compared with their anti-CENP-B–negative patients, anti-CENP-B–positive SLE patients displayed a markedly divergent therapeutic response.

**Conclusions:**

Anti-CENP-B positivity defines a distinct SLE subset characterized by older age at onset, milder renal involvement, Raynaud’s phenomenon, and specific humoral alterations; importantly, these patients also show a treatment response that differs significantly from that of the anti-CENP-B-negative group, underscoring the imperative for personalized, precision therapy.

## 1 Introduction

Systemic lupus erythematosus (SLE) is a prototypic autoimmune disease characterized by aberrant activation of autoreactive T and B cells, resulting in diverse clinical manifestations and the production of a wide array of autoantibodies (1, 2). These autoantibodies serve as biomarkers that not only help identify individuals at risk of developing SLE, predict disease progression, organ damage, prognosis, and treatment response, but also elucidate the relationship between specific antibodies and the heterogeneity of clinical manifestations (3). The presence of antinuclear antibodies (ANA) at diagnosis is nearly universal, with very few exceptions (4). Importantly, a positive ANA test is a prerequisite for classifying a patient as having SLE according to the 2019 classification criteria established by the American College of Rheumatology (ACR) and the European League Against Rheumatism (EULAR) (5).

In clinical testing, the extractable nuclear antigen (ENA) antibody profile is a crucial component of ANA testing. The most commonly targeted antigens in ENA panels include dsDNA, U1-nRNP/Sm, Sm, SSA/Ro60, Ro52, SSB/La, Scl-70, Jo-1, Centromere-B, histones, nucleosomes, and Ribosomal P (6). Among these, anti-Sm and anti-dsDNA antibodies are well-established hallmarks of SLE and are included in the ACR classification criteria for SLE (5, 7, 8). In addition to these classic autoantibodies, SLE patients often present with other autoantibodies. Recent studies suggest that different autoantibodies may be associated with distinct clinical manifestations of the disease (9).

Anti-centromere antibodies (ACA) primarily target three centromere proteins: CENP-A, B, and C, with CENP-B serving as the predominant target. CENP-B is an 80-kDa protein that binds DNA via its N-terminal domain (10). This was reflected in the uniformly high CENP-B band intensity observed across multiple patient sera, in contrast to the variable intensities seen for CENP-A and CENP-C. Furthermore, no anti-CENP-B autoantibodies were detected in ACA-negative patients (11). Historically, ACA has been a serological hallmark of the limited cutaneous form of systemic sclerosis (lcSSc), previously termed CREST syndrome (calcinosis, Raynaud’s phenomenon, esophageal dysmotility, sclerodactyly, and telangiectasias) (12). More recently, a high prevalence of anti-CENP-B antibodies has been reported in primary biliary cholangitis (PBC) and primary Sjögren’s syndrome (pSS), with associations also noted in lymphoma (13, 14). In contrast, data on ACA or anti-CENP-B in SLE remain limited. Studies indicate an ACA prevalence of approximately 2% to 11% in SLE cohorts (15–17). While some autoantibodies bind directly to DNA or nucleosome proteins, others target RNA-binding proteins (RBPs), forming pathogenic immune complexes via tissue deposition or cytokine induction (18). The specific role of anti-CENP-B in SLE remains largely unexplored, warranting further investigation to determine whether anti-CENP-B-positive SLE patients exhibit features overlapping with other autoimmune diseases, particularly systemic sclerosis. While anti-CENP-B has been extensively characterized as a key autoantibody in several other autoimmune diseases, its role in SLE continues to be debated and insufficiently explored, owing largely to inconsistencies in study design, variability in patient stratification criteria, and a lack of longitudinal data linking this autoantibody to specific SLE clinical phenotypes or outcomes.

Therefore, this study aims to delineate the associations of specific clinical manifestations, immune cell profiles, biochemical markers, treatment response and risk factors with anti-CENP-B seropositivity in SLE. The findings are expected to enable a more precise subclassification of SLE and ultimately inform improved diagnostic and clinical management strategies.

## 2 Materials and methods

### 2.1 Patients’ inclusion and exclusion criteria

**Figure 1:**
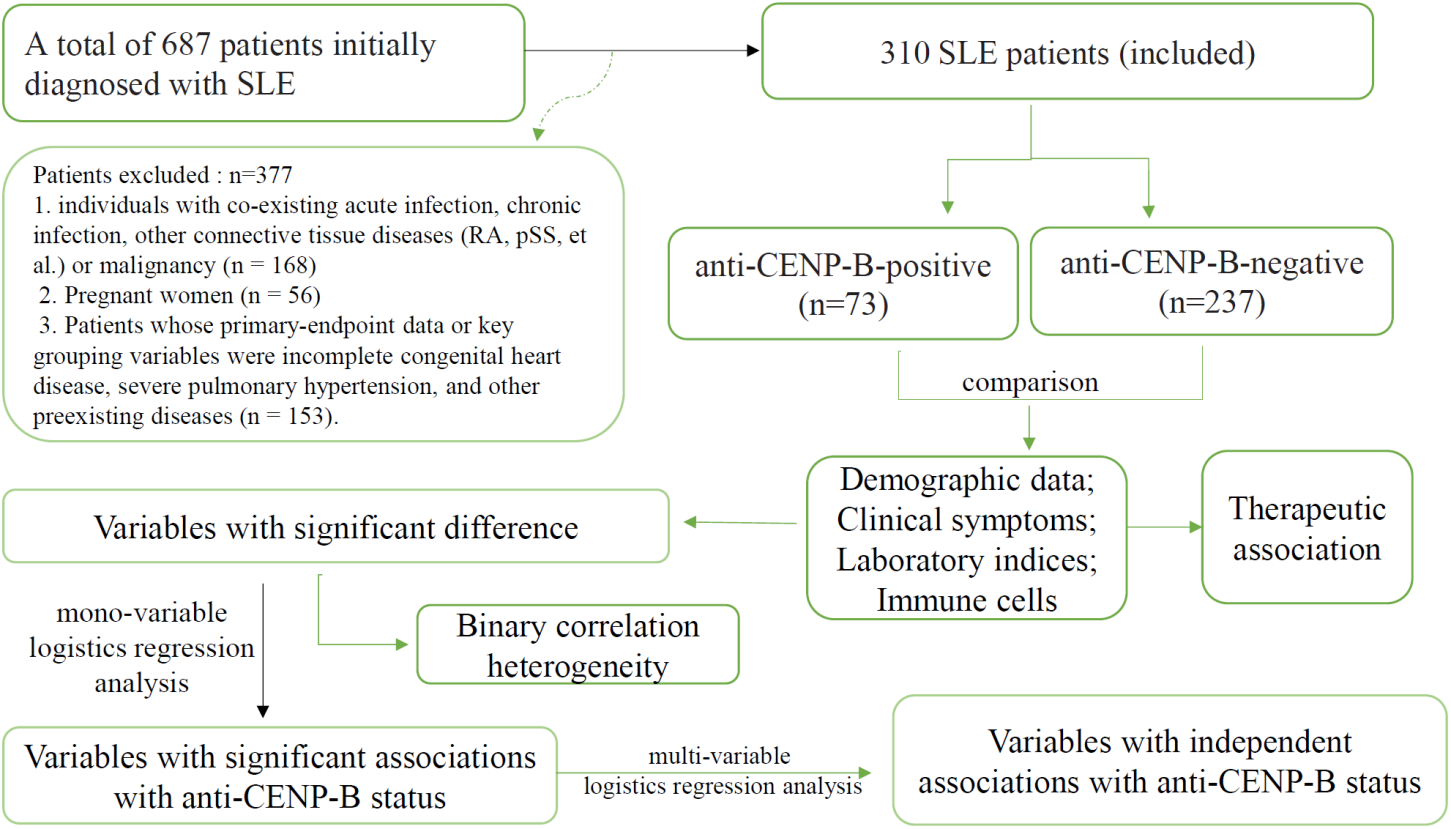
The flowchart of this study.

This retrospective analysis was conducted on consecutive cases of systemic lupus erythematosus (SLE) diagnosed at Henan Provincial People’s Hospital, Zhengzhou, China, between March 2023 and May 2025. The study protocol was approved by the hospital’s Ethics Committee (approval no. 2024-96), and all procedures were performed in accordance with the Declaration of Helsinki. A total of 687 patients who met either the 2019 EULAR/ACR classification criteria for SLE (5) or the 2012 SLICC criteria (19) were included. To mitigate potential confounding effects on SLE-related clinical and immunologic indices, we sequentially excluded (**Figure 1**): (1) 168 individuals with co-existing acute infection, chronic infection, other connective tissue diseases (RA, pSS, et al) or malignancy; (2) 56 pregnant women, in whom gestation-associated immune modulation could bias results; and (3) 153 subjects whose primary-endpoint data or key grouping variables were incomplete. After these exclusions, 310 eligible SLE patients remained and were stratified by anti-CENP-B antibody status into seropositive (anti-CENP-B positive) and seronegative (anti-CENP-B negative) cohorts for all subsequent analyses. All patients included in this study were newly diagnosed with SLE and had no prior history of SLE-specific treatment. The follow-up timeline was defined as follows: M0 represented the baseline assessment at the initiation of treatment, and M6 indicated the follow-up assessment at 6 months after treatment initiation.

### 2.2 Demographic and clinical data collection

Demographic characteristics, including gender and age, as well as clinical manifestations—such as constitutional, mucocutaneous, musculoskeletal, renal, hematologic, neuropsychiatric, gastrointestinal, cardiac, and pleuropulmonary involvement, along with Raynaud’s phenomenon—were retrospectively extracted from patients’ electronic medical records. All of organ involvements were defined according to the corresponding items in the SLICC-2012 classification criteria and the EULAR/ACR-2019 criteria (5, 19), supplemented by standard clinical investigations. All assessments were independently reviewed by two rheumatologists; disagreements were resolved by a senior third reviewer. Lupus nephritis (LN) is a common and severe manifestation of SLE. To examine the relationship between LN subtypes and anti-CENP-B status, data on LN occurrence were collected. LN subtypes were classified according to the WHO/ISN criteria (20). Renal involvement was established either by renal biopsy or, in the absence of histological confirmation, by the presence of overt renal manifestations (proteinuria ≥0.5g/L or active urinary sediment) documented during a lupus flare (21). Treatment modalities included standard of care (SOC) alone, or SOC combined with biological agents (belimumab, telitacicept, or rituximab). SOC consisted of corticosteroids and hydroxychloroquine or chloroquine, with or without additional immunosuppressants such as azathioprine, methotrexate, cyclosporine A, mycophenolate mofetil, cyclophosphamide, or sulfasalazine. All treatment regimens were selected in accordance with 2020 Chinese Guidelines for the Diagnosis and Treatment of Systemic Lupus Erythematosus (22) or 2025 Chinese guidelines for the diagnosis and treatment of systemic lupus erythematosus (23).

### 2.3 Laboratory indices

Disease activity was evaluated using the Systemic Lupus Erythematosus Disease Activity Index 2000 (SLEDAI-2K) (24). Serum anti-double-stranded DNA (anti-dsDNA) antibody levels were quantified using enzyme-linked immunosorbent assay (ELISA; Euroimmun AG, Lübeck, Germany). Serum was deemed ANA-positive when characteristic indirect immunofluorescence (IIF) staining was present at an initial dilution of 1:100. Antibody titers were scored semi-quantitatively (1+ – 4+) according to endpoint reactivity at dilutions of 1:100, 1:320, 1:1,000 and ≥1:3,200. Autoantibodies to extractable nuclear antigens (anti-U1-nRNP/Sm, anti-Sm, anti-Ro52, anti-SSA, anti-SSB, anti-nucleosome, anti-histone, anti-ribosomal P, anti-Scl-70, anti-Jo-1 and anti-CENP-B) were determined by line-blot immunoassay (EUROLINE kits: EUROLINE ANA Profile1, Euroimmun AG, Lübeck, Germany). Interpretation of the specific autoantibody profile was performed with EUROLineScan software (EUROIMMUN). Each nitrocellulose strip incorporates an internal control band; intense chromogenic reactivity at this locus validates technical proficiency. Antigen-coated positions exhibiting absence of staining (i.e., a white band) were classified as negative. In accordance with the manufacturer’s specifications, a grey-scale optical density ≥ 11 arbitrary units at any antigen-coated site was defined as the threshold for positivity, contingent upon concurrent demonstration of a clearly discernible control band. Positive and negative calibrators supplied with the assay were analyzed in parallel with every sample batch to ensure run integrity. Serum IgG, IgM, IgA, C3 and C4 were quantified by immunoturbidimetry on a Roche Cobas 6000 E501 analyzer. 24-hour urinary total protein (24h-UTP) was measured with the pyrogallol red–molybdate method on an Abbott ARCHITECT c8000 analyzer. All assays were performed according to standard laboratory protocols.

### 2.4 Flow cytometry analysis

Peripheral blood was collected into EDTA tubes from all enrolled patients, and T-cell subsets (CD3^+^, CD4^+^, CD8^+^), CD19^+^ B cells and CD16^+^CD56^+^ NK cells were analyzed on a Wmini5268 flow cytometer (Guangzhou Weimi Bio-Tech Co., Ltd.) following the manufacturer’s instructions. After gating on live cells with FSC/SSC to exclude debris, lymphocytes were defined as CD45^+^ events with low SSC and intermediate FSC; CD3^+^ T cells were then identified and subdivided into CD4^+^ and CD8^+^ subsets, while B cells were gated as CD3^-^CD19^+^ and NK cells as CD3^-^CD16^+^CD56^+^. Fluorescence-minus-one (FMO) and isotype controls were used to set gates and avoid false signals. The antibody panel consisted of APC-Cy7-CD45-Ab, FITC-CD3-Ab, APC-CD4-Ab, PerCP-CD8-Ab, APC-CD19-Ab and PE-CD16/CD56-Ab. Lymphocyte subsets were compared between anti-CENP-B–positive and anti-CENP-B–negative SLE patients.

Peripheral lymphocyte subsets, immunoglobulin and complement levels, and disease-activity indices including SLEDAI-2K anti-dsDNA antibodies, and 24-hour urinary total protein (24h-UTP) were assessed in 310 SLE patients at baseline (M0) and after six months of therapy (M6). Each group was further subdivided by treatment regimen: standard of care (SOC) or SOC plus biologics.

### 2.5 Statistical analysis

This study focused on contrasting the baseline (M0) characteristics between the anti-CENP-B–negative and anti-CENP-B–positive groups. To evaluate the impact of anti-CENP-B positivity on therapy, we also compared the treatment responses (M6 vs. M0) achieved in these two cohorts. Statistical analyses were performed with SPSS 23.0 (IBM Corp., Armonk, NY, USA). Figures were prepared in GraphPad Prism 10.1.2 (GraphPad Software, San Diego, CA, USA). Logistic-regression models and associated visualizations were constructed in R 4.3.1 (R Foundation for Statistical Computing, Vienna, Austria). The Continuous variables were compared between groups with the Mann–Whitney U test or Wilcoxon test. Categorical data are reported as counts (percentages) and were analyzed with the Chi-square test or Fisher’s exact test, as appropriate. Correlations between two continuous variables were quantified with Spearman’s rank correlation coefficient. Two-tailed *p*<0.05 were considered statistically significant. Variables associated with anti-CENP-B positivity at *p*<0.05 in univariate logistic regression were entered into a multivariable logistic-regression model to identify independent predictors among patients with anti-CENP-B positive SLE. A nomogram based on the final multivariable model was developed to provide a graphical representation of the predictors of anti-CENP-B positivity.

## 3 Results

### 3.1 Distinct clinical characteristics of SLE patients with different anti-CENP-B antibody status

**Table 1** summarizes the baseline characteristics and clinical outcomes of the 310 patients with SLE included in the analyses. Among them, 73 patients (23.54%) were anti-CENP-B-positive and 237 (76.46%) were anti-CENP-B-negative. Both groups were predominantly female (91.78% vs 89.87%, *p* > 0.05). Anti-CENP-B-positive patients were significantly older than anti-CENP-B-negative patients (median 49 years vs. 33 years, *p*<0.05). With respect to clinical manifestations, Raynaud’s phenomenon (20.55% vs 4.64%, *p*<0.01), cardiac involvement (5.48% vs 0.42%, *p*<0.01) and pleuropulmonary involvement (10.96% vs 4.64%, *p*<0.05) occurred more frequently in the anti-CENP-B-positive cohort. In contrast, renal involvement was less common in this group (36.98% vs 51.47%, *p*<0.001). No statistically significant differences were observed for constitutional, mucocutaneous, musculoskeletal, hematological, neuropsychiatric or gastrointestinal manifestations (all *p* > 0.05).

**Table 1:** Clinical and demographic characteristics of SLE patients.

Variable	Anti-CENP-B-N	Anti-CENP-B-P	p-value
	N=237 (76%)	N=73 (24%)	
Gender			0.798
Female	213 (89.87%)	67 (91.78%)	
Male	24 (10.13%)	7 (8.22%)	
Age (median, IQR)	33.00 [24.00, 44.00]	49.00 [36.00, 54.00]	<0.001
Clinical manifestations			
Constitutional	65 (27.43%)	20 (27.40%)	>0.999
Mucocutaneous	79 (33.33%)	29 (39.73%)	0.316
Musculoskeletal	76 (32.07%)	28 (38.36%)	0.32
Renal involvement	122 (51.47%)	27 (36.98%)	<0.001
Hematological	26 (10.97%)	5 (6.85%)	0.305
Raynaud phenomenon	11 (4.64%)	15 (20.55%)	<0.001
Neuropsychiatric	20 (8.44%)	4 (5.48%)	0.408
Digestive	20 (8.44%)	6 (8.22%)	0.953
Cardiac	1 (0.42%)	4(5.48%)	0.003
Pleuropulmonary	11 (4.64%)	8 (10.96%)	0.049

Age was compared with the Mann–Whitney U test, others were compared with the χ² test or Fisher’s exact test.

### 3.2 Lupus nephritis and anti-CENP-B antibody status

**Figure 2:**
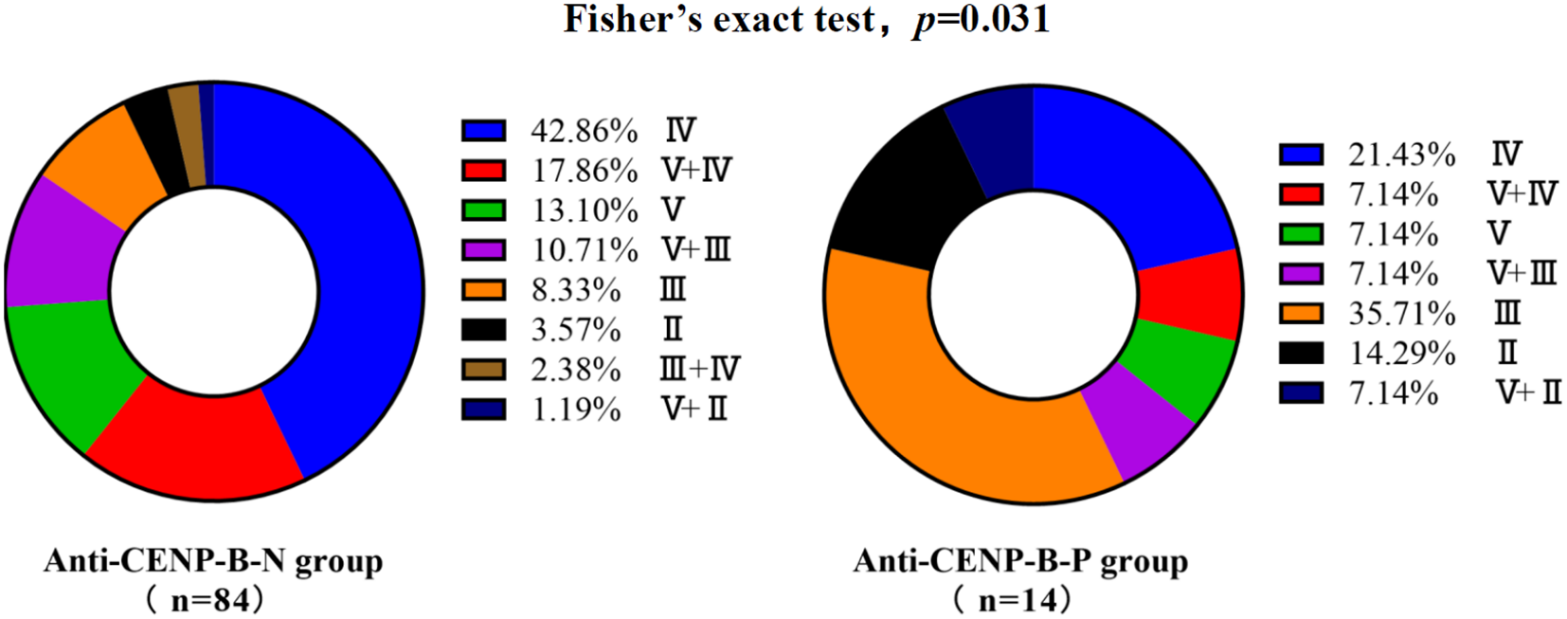
Lupus nephritis distribution with different anti-CENP-B antibody status. Bar heights indicate the percentage of each class; combined classes are denoted as “primary + secondary” (e.g., V+IV, V+III, V+II). Fisher’s exact test was used and *p*<0.05 was considered statistically significant.

Renal biopsy was performed at clinical onset in 84 of 115 anti-CENP-B-N LN cases and in 14 of 17 anti-CENP-B-P LN cases. LN incidence was significantly lower in anti-CENP-B-positive (anti-CENP-B-P) patients than in anti-CENP-B-negative (anti-CENP-B-N) patients (23.29% vs. 48.52%, *p*<0.01). Among anti-CENP-B-N biopsies, the most common WHO/ISN classes were class IV (42.86%), class V+IV (17.86%) and class V (13.10%) (**Figure 2**). In contrast, anti-CENP-B-P biopsies most frequently exhibited class III (35.71%), followed by class IV (21.43%) and class II (14.29%) (**Figure 2**). Fisher’s exact test demonstrated a significant difference in histological class distribution between the two groups (*p*=0.031), indicating that anti-CENP-B seropositivity is associated with a shift toward less advanced proliferative lesions (class III) and away from mixed/proliferative patterns (V + IV) in LN.

### 3.3 Differential autoantibody profiles between anti-CENP-B-positive and anti-CENP-B-negative SLE patients

**Figure 3:**
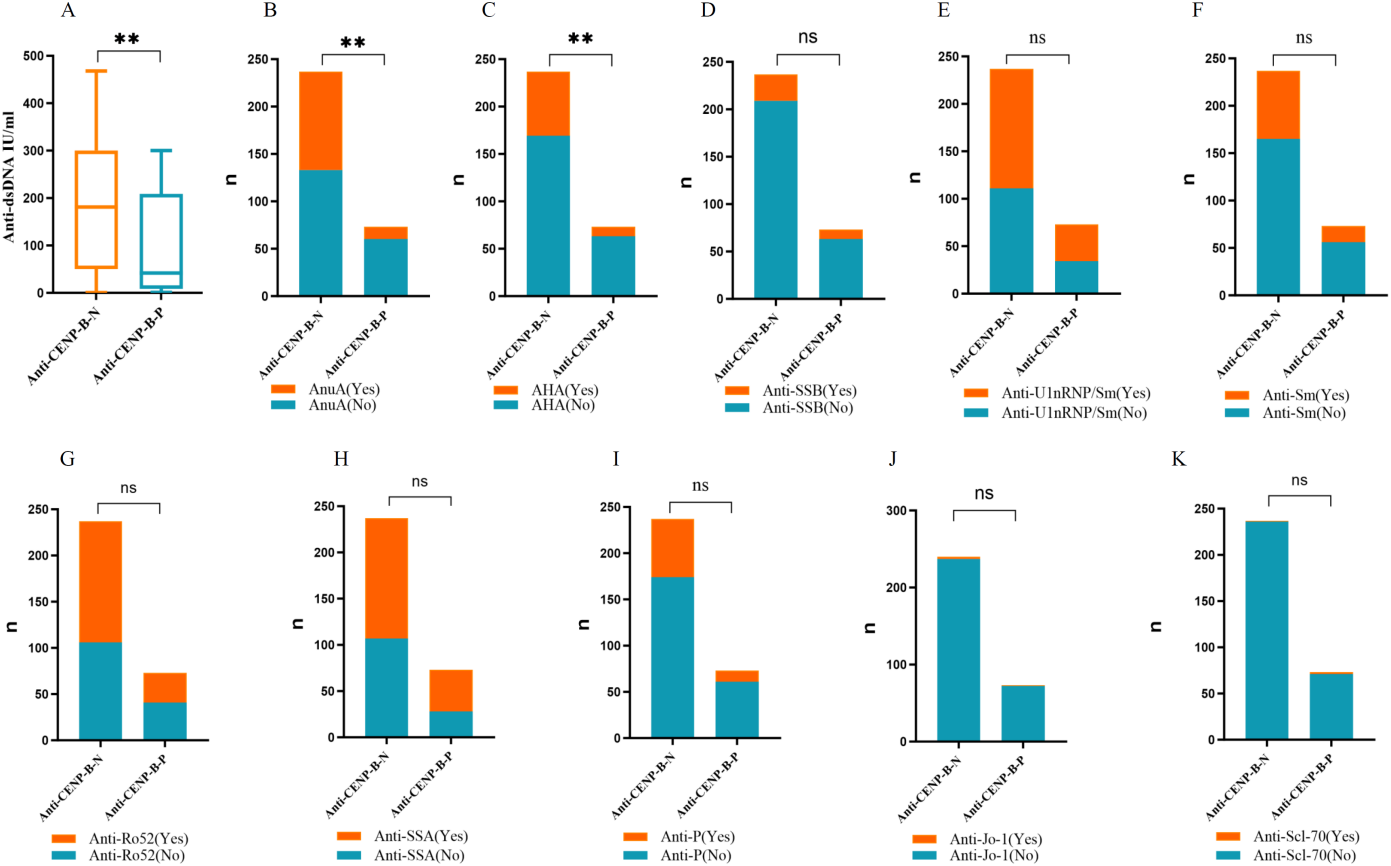
The comparisons of the autoantibody levels between anti-CENP-B -negative and anti-CENP-B -positive groups. **(A)** The comparisons of anti-dsDNA levels between the two SLE groups. **(B–K)** The comparisons the occurrences of AnuA, AHA, anti-SSB, anti-U1-nRNP/Sm, anti-Sm, anti-Ro52, anti-SSA, anti-P, anti-Jo-1 and anti-Scl-70 between the two SLE groups. *, p<0.05; **, p<0.01; ns, no significance. Mann–Whitney U test **(A)** and Chi-square test **(B–K)** were used for analyses and *p*<0.05 was considered to be statistically significant.

All 310 patients were ANA-positive. ANA titers differed significantly between the anti-CENP-B-P and anti-CENP-B-N cohorts (**Supplementary Figure S1**). High-titer ANA (≥ 1:1,000) was present in 72.15% of anti-CENP-B-N patients, whereas the majority of anti-CENP-B-P subjects displayed intermediate titers (1:320–1:1,000). Distinct autoantibody reactivities were also observed. anti-dsDNA levels were significantly elevated in the anti-CENP-B-N group (**Figure 3A**). Likewise, positivity for AnuA (43.9% vs. 17.8%, *p*<0.05) and AHA (28.7% vs. 13.7%, *p*<0.05) antibodies was more frequent in anti-CENP-B-N patients (**Figures 3B, C**). In contrast, the prevalence of ENA specificities—including anti-SSB, anti-U1-RNP/Sm, anti-Sm, anti-Ro52, anti-SSA, anti-P, anti-Scl-70, and anti-Jo-1—did not differ significantly between the two groups (**Figures 3D–K**).

### 3.4 Differential laboratory markers between anti-CENP-B-positive and anti-CENP-B-negative SLE cases

**Figure 4:**
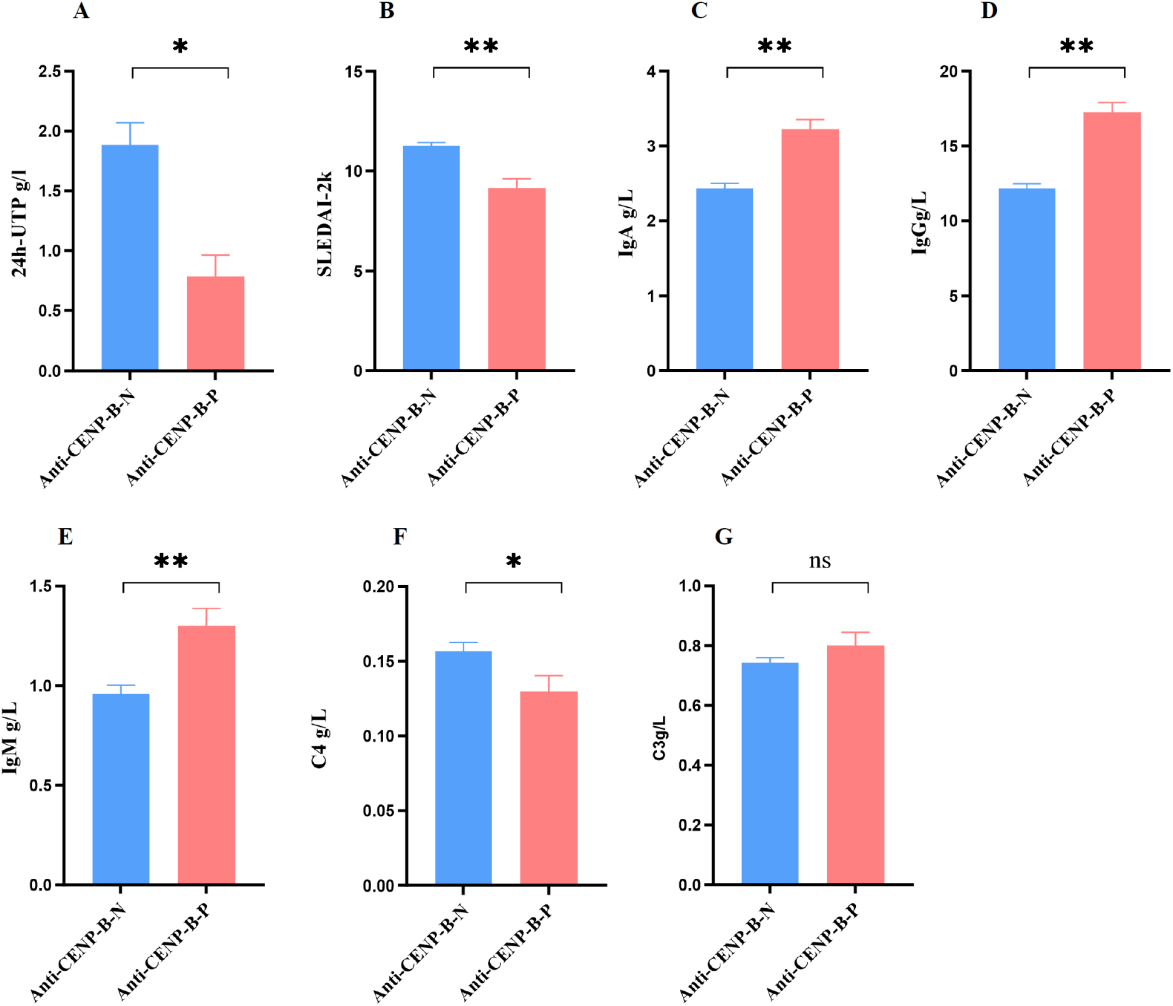
Comparisons of activity index (SLEDAI-2k) and laboratory markers between anti-CENP-B-negative and anti-CENP-B-positive SLE patients. **(A-B)** The significant difference of 24h-UTP and SLEDAI-2k between SLE subgroups. **(C–G)** The comparisons of immunoglobulin (IgA, IgG, and IgM) and complement (C4 and C3) between anti-CENP-B-negative group and anti-CENP-B-positive group. *, *p*<0.05; **, *p*<0.01; ns, no significant difference. The Mann-Whitney U test was used and *p*<0.05was considered significant.

In line with the higher prevalence of LN among anti-CENP-B-negative patients, 24h-UTP and SLEDAI-2K scores were significantly elevated in this group (**Figures 4A, B**). Conversely, serum immunoglobulin levels (IgA, IgG and IgM) were markedly higher in anti-CENP-B-positive patients (**Figures 4C-E**), whereas complement C4 was significantly reduced (**Figure 4F**). No inter-group difference was detected for complement C3 (**Figure 4G**).

### 3.5 Differential lymphocytes profiles between anti-CENP-B-negative and anti-CENP-B-positive patients

**Figure 5:**
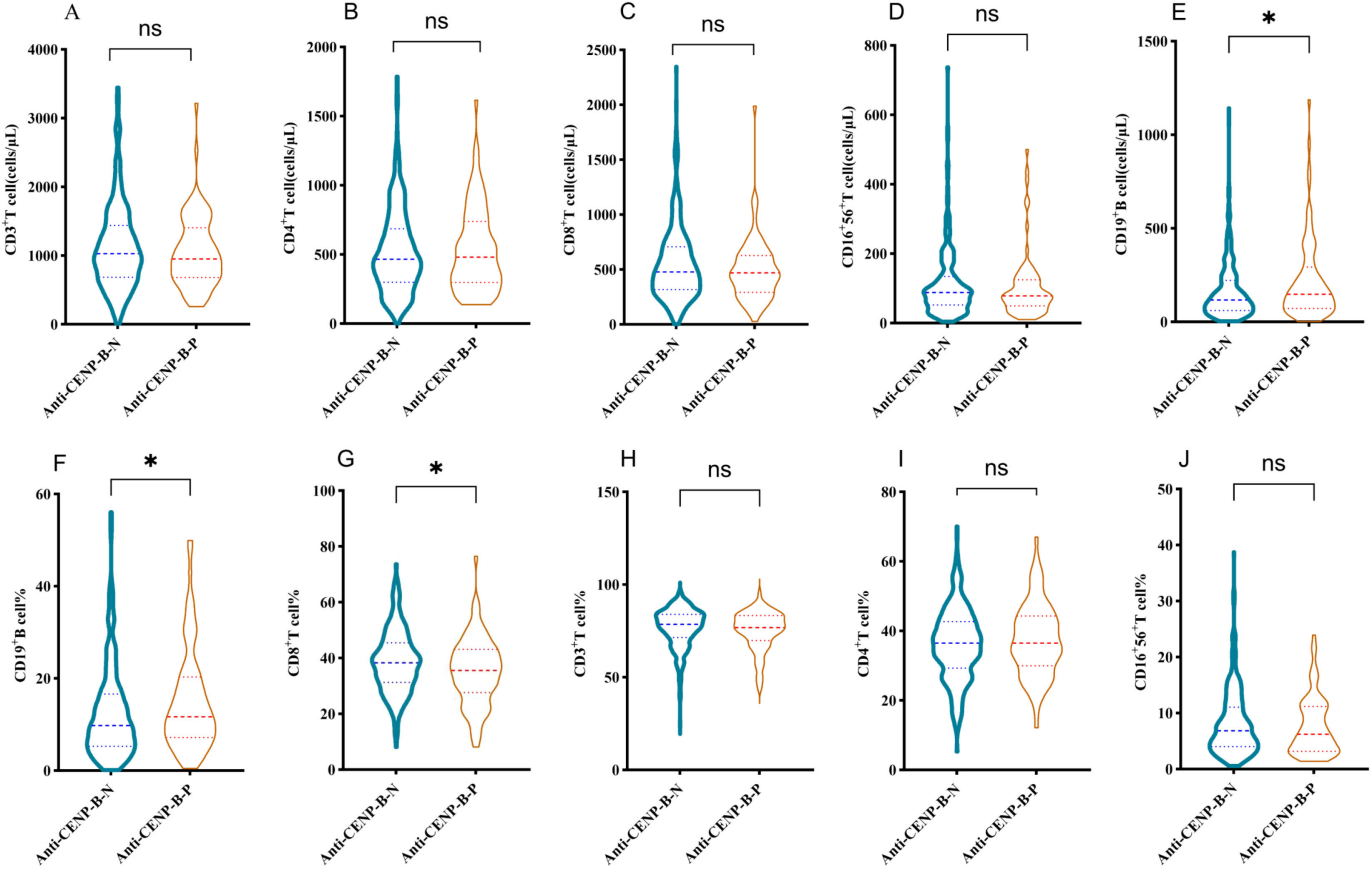
Differences in lymphocyte subset distributions between anti-CENP-B-negative and anti-CENP-B-positive patients. **(A-D)** Comparisons of the distributions of CD3^+^T cells, CD4^+^T cells, CD8^+^T cells, and CD16^+^CD56^+^NK cells between the two groups. **(E-G)** Significant differences in the absolute counts of CD19^+^ B cells, the percentage of CD19^+^ B cells, and the percentage of CD8^+^T cells between the two groups. **(H-J)** Comparisons of the percentages of CD3^+^T cells, CD4^+^T cells, and CD16^+^56^+^NK cells between the two groups. *p<0.05; ns, no significant difference. The Mann-Whitney U test was used and *p*<0.05 was considered statistically significant.

As illustrated in **Figure 5**, absolute numbers of CD3^+^ T, CD4^+^ T, CD8^+^ T and CD16^+^CD56^+^ NK cells did not differ between anti-CENP-B-negative and anti-CENP-B-positive patients (**Figures 5A–D**). In contrast, both the absolute count and the proportion of CD19^+^ B cells were significantly elevated in the anti-CENP-B-positive group (**Figures 5E, F**), whereas the percentage of CD8^+^ T cells was reduced (**Figure 5G**). No significant inter-group differences were observed in the relative proportions of CD3^+^ T, CD4^+^ T or CD16^+^CD56^+^ NK cells (**Figures 5H–J**).

### 3.6 Correlations of CD19^+^B cell and CD8^+^ T cell with clinical-biological parameters

**Figure 6:**
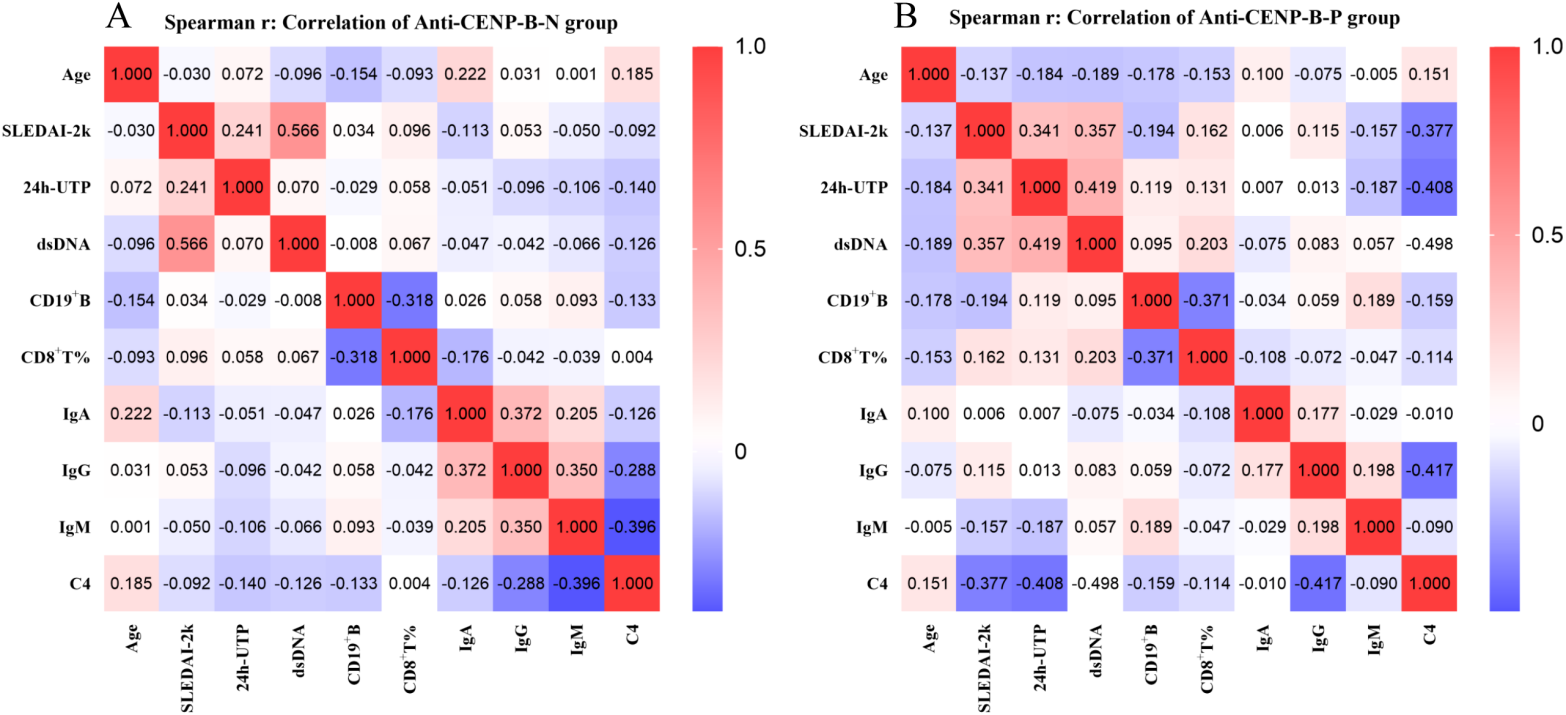
Spearman correlations between clinical parameters in anti-CENP-B-negative **(A)** and anti-CENP-B-positive SLE patients **(B)**. Red and blue colors represent positive and negative correlations, respectively. The color intensity reflects the strength of the correlation (−1 to 1), as indicated by the scale bar. *P*<0.05 was considered statistically significant.

To elucidate the clinical significance of B cells and T cell subgroups, we examined their correlations with key clinical parameters, including age, SLEDAI-2K, 24h-UTP, anti-dsDNA, IgA, IgG, IgM, and C4 levels. **Figure 6** summarizes pairwise correlations. In both cohorts, SLEDAI-2K scores presented low to moderate positive correlations with 24h-UTP and anti-dsDNA (anti-CENP-B-N: *r*=0.241 and 0.566; anti-CENP-B-P: *r*=0.341 and 0.357; all *p*<0.05). A moderate correlation was also observed between 24h-UTP and anti-dsDNA in anti-CENP-B-positive patients (*r*=0.419, *p*<0.01), whereas no such correlation was detected in the negative group. C4 showed moderate negative correlations with SLEDAI-2K scores, anti-dsDNA, and IgG in anti-CENP-B-positive patients (*r*=–0.377 to –0.498, *p*<0.01), whereas in anti-CENP-B-negative patients, it additionally presented a low negative correlation with CD19^+^ B-cell counts (*r*=–0.133, *p*<0.05) and a moderate negative correlation with IgM (*r*=–0.396, *p*<0.05). CD19^+^ B-cell abundance was inversely correlated with CD8^+^ T-cell percentage in both groups. Age exhibited no significant correlations with the other measured parameters; instead, it was only correlated with IgA and C4 in anti-CENP-B-negative patients, with both associations representing low positive correlations (IgA: *r*=0.222, *p*<0.05; C4: *r*=0.185, *p*<0.05). These divergent correlation matrices underscore distinct immunological phenotypes, whereas shared IgG–IgM covariation reflects expected immunoglobulin collinearity.

### 3.7 Logistic regression analysis of independent indicators of anti-CENP-B-positive SLE patients

**Figure 7:**
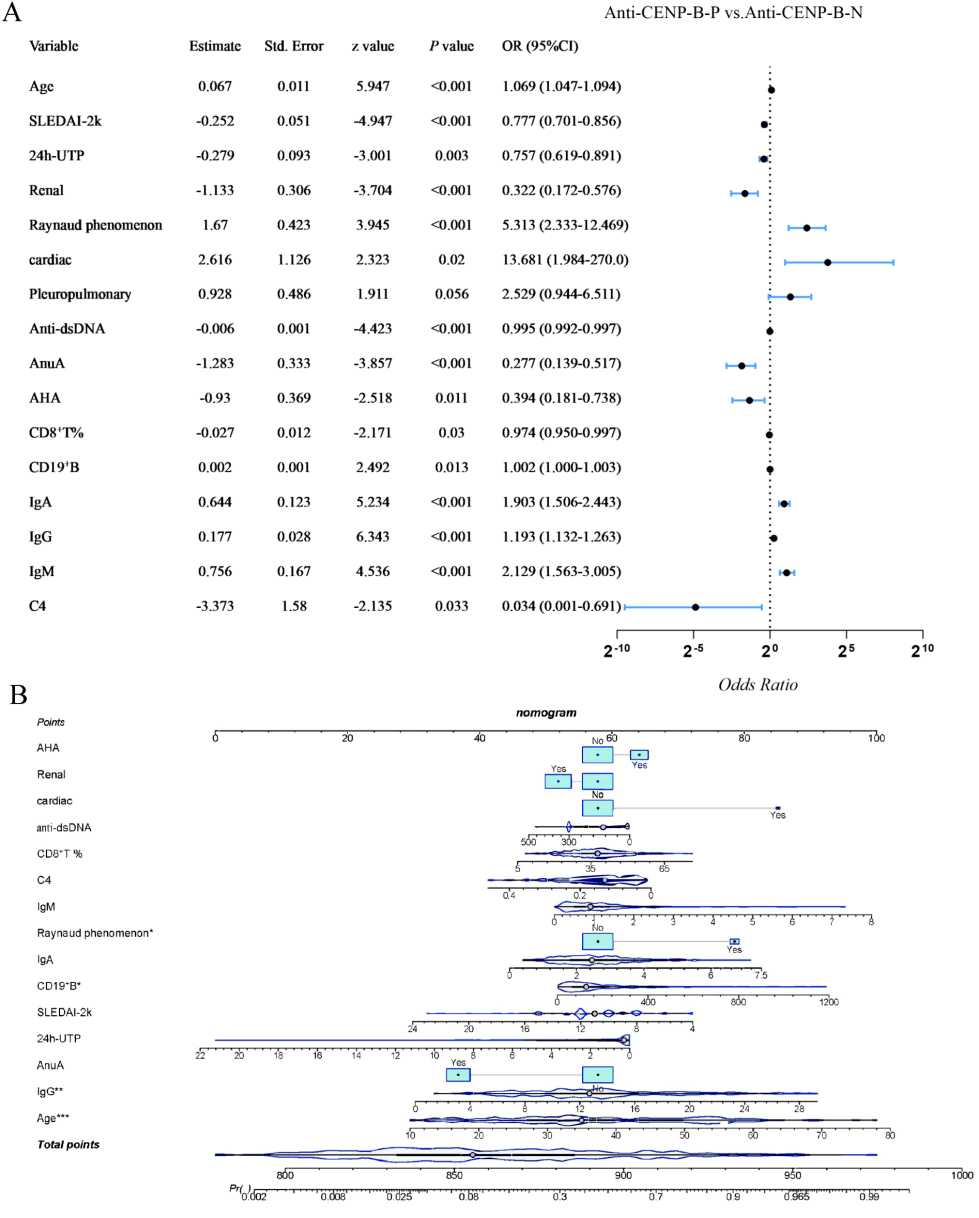
Univariate and Multivariate logistic regression analysis of differential variables with anti-CENP-B status in SLE patients. *, *p*<0.05; **, *p*<0.01; ***, *p*<0.001. Odds Ratio >1 and Odds Ratio <1 represent positive and negative correlations, respectively, and *p*<0.05 was considered statistically significant.

Univariate logistic regression was performed on all variables that differed between groups (*p*<0.05) to identify potential confounders. After exclusion of pleuropulmonary involvement (*p* > 0.05), the remaining factors retained a significant association with anti-CENP-B positivity (*p*<0.05, **Figure 7A**). Renal involvement, SLEDAI-2K score, 24-h UTP, anti-dsDNA, AnuA, AHA, CD8^+^ T-cell percentage and C4 were inversely related (*OR*<1), whereas age, cardiac involvement, Raynaud’s phenomenon, CD19^+^ B-cell count, IgA, IgG and IgM showed positive associations (*OR* > 1). Variables retaining significance in the univariate screen (p<0.05) were entered into a multivariate logistic regression model, and a nomogram was derived (**Figure 7B**). Age, Raynaud’s phenomenon, absolute CD19^+^ B-cell count and IgG concentration remained independently associated with anti-CENP-B positivity (all *OR* > 1, *p*<0.05). The model’s performance was good, with an AUC of 0.896 (95%CI: 0.854–0.938, *p*<0.001; **Supplementary Figure S2**).

### 3.8 Anti-CENP-B-positive SLE patients exhibit a distinct clinical treatment response

Regardless of whether the SLE patients were anti-CENP-B–positive or –negative, and regardless of whether they received the SOC regimen or the SOC + biologics (belimumab, telitacicept and rituximab) regimen, all disease-activity indices—SLEDAI-2K score, anti-dsDNA antibody level and 24h-UTP declined significantly (all *p*<0.05, **Tables 2**, **3**). However, at the immunomodulatory-molecule level (lymphocyte subsets, immunoglobulins and complement), the two treatment strategies and the different disease groups exhibited clearly divergent responses. After 6 months of SOC therapy, the absolute counts of CD3^+^T, CD3^+^CD4^+^T, CD3^+^CD8^+^T, CD19^+^B and CD3^-^CD16^+^CD56^+^ cells remained stable in anti-CENP-B–negative SLE patients (**Table 2**), whereas the percentage of CD19^+^ B cells fell significantly (*p*=0.005). By contrast, in the anti-CENP-B–positive cohort, the absolute number and proportion of CD19^+^ B cells fell sharply after therapy (*p*<0.001). In contrast, the absolute counts and percentages of CD3^+^ T cells and CD3^-^CD16^+^CD56^+^ NK cells, together with the absolute counts of CD3^+^CD4^+^ and CD3^+^CD8^+^ T cells, all rebounded markedly (all *p*<0.05). Yet the proportions of CD3^+^CD4^+^ and CD3^+^CD8^+^ T cells, though numerically higher, remained statistically similar to baseline (*p* > 0.05). Humoral indices improved modestly in the anti-CENP-B–negative subgroup, with only C3 increasing (*p*=0.004), whereas the anti-CENP-B–positive group showed significant recovery of both C3 and C4 (*p*<0.05) and a pronounced decrease in IgG (*p*=0.001) and IgM (*p*=0.049).

**Table 2:** Detailed characteristics of anti-CENP-B-positive and anti-CENP-B-negative SLE patients under SOC treatment.

Feature	Anti-CENP-B-Negative			*P*	Anti-CENP-B-Positive			*P*
	M0 (median, IQR)	M6 (median, IQR)	M6-M0 (median, IQR)		M0 (median, IQR)	M6 (median, IQR)	M6-M0 (median, IQR)	
SLEDAI-2K	12 (9.5-12)	8 (6-10)	-4 (-4--2)	<0.001	8 (5-10)	4.5 (4-6)	-2 (-4--2)	<0.001
24h-UTP	0.23 (0.06-1.72)	0.07 (0.03-0.97)	-0.04 (-0.68-0)	0.008	0.16 (0.08-0.4)	0.06 (0.03-0.14)	-0.07 (-0.23--0.04)	<0.001
dsDNA (IU/ml)	139.14 (48.75-300)	24.37 (7.56-53.88)	-82.8 (-219.06--22.51)	<0.001	15.8 (7.82-98)	10 (5.2-39.59)	-6.25 (-48.44--1.95)	<0.001
CD16 + 56+NK	85 (49-135.5)	102 (54.5-173.5)	15 (-17.5-44.5)	0.064	82 (49.25-132)	136.5 (86.75-243.5)	45 (4.25-99.25)	<0.001
CD16 + 56+NK%	7.76 (5.09-12.38)	8.34 (4.18-11.18)	0.22 (-2.31-2.43)	0.740	6.59 (3.26-11.51)	10.87 (6.79-15.22)	2.69 (0.16-6.02)	<0.001
CD19+B	104 (57.5-225.5)	124 (53.5-184.5)	6 (-71-55.5)	0.648	147 (76.65-234)	96.5 (48.5-137)	-47.65 (-116.35--0.28)	<0.001
CD19+B%	9.56 (5.89-18.71)	7.94 (3.84-14.45)	-1.67 (-7.12-1.29)	0.005	11.56 (7.24-18.63)	6.16 (3.36-9.84)	-4.15 (-10.49--1.52)	<0.001
CD3+CD4+T	358 (230.5-492.5)	454 (307-675.5)	38 (-65-228)	0.080	480.5 (296.5-756)	558 (405.75-747)	89.5 (-21.25-210)	0.004
CD3+CD4+T%	33.9 (26.9-39.34)	35.57 (28.88-40.14)	1.84 (-3.63-4.94)	0.305	36.74 (31.72-43.88)	38.12 (32.6-46.99)	0.71 (-4.91-6.88)	0.679
CD3+CD8+T	389 (285-694)	531 (342.5-771.5)	55 (-143.5-240)	0.219	468 (300.75-591.5)	529.5 (397.25-683)	78.5 (-58.5-200)	0.019
CD3+CD8+T%	36.9 (30.93-45.73)	42.05 (32.44-49.4)	0.46 (-3.48-7.39)	0.153	34.88 (28.52-43.04)	37.06 (32.02-42.32)	1.62 (-3.17-6.77)	0.059
CD3+T	867 (549.5-1252.5)	1011 (750.5-1477.5)	121 (-175-548)	0.081	974 (681.25-1411.75)	1150 (839-1510.5)	123 (-57.25-373)	0.007
CD3+T%	77.05 (70.75-83.01)	78.92 (72.05-86.5)	1.39 (-4.14-8.21)	0.107	76.76 (70.74-81.62)	79.16 (72.34-84.27)	1.63 (-3-8.09)	0.037
IgA(g/L)	2.52 (1.77-3.09)	2.52 (1.64-3.16)	-0.14 (-0.42-0.22)	0.195	3.15 (2.37-3.86)	2.8 (2.12-3.76)	-0.3 (-0.9-0.3)	0.068
IgG(g/L)	12.26 (8.9-14.5)	12.07 (9.23-14.8)	-0.13 (-3.33-2.03)	0.531	15.36 (12.82-21.88)	13.56 (11.22-15.45)	-2.68 (-8.34-0.31)	<0.001
IgM(g/L)	0.96 (0.5-1.27)	0.88 (0.54-1.25)	-0.03 (-0.23-0.09)	0.132	1.39 (0.92-1.75)	1.21 (0.8-1.74)	-0.16 (-0.58-0.22)	0.049
C3(g/L)	0.77 (0.56-0.88)	0.85 (0.66-1.17)	0.17 (-0.06-0.28)	0.004	0.85 (0.56-1.17)	1.08 (0.88-1.29)	0.23 (-0.04-0.51)	<0.001
C4(g/L)	0.14 (0.1-0.24)	0.19 (0.11-0.25)	0.03 (-0.03-0.08)	0.060	0.13 (0.05-0.2)	0.21 (0.16-0.27)	0.08 (0.02-0.16)	<0.001

For the variables, the median values with interquartile range (IQR) were provided. SOC, standard of care; M0, baseline (pre-treatment); M6, six-month post-treatment. Paired Wilcoxon test was used for comparison and *p*<0.05 was considered statistically significant.

**Table 3:** Detailed characteristics of anti-CENP-B-positive and anti-CENP-B-negative SLE patients under SOC + biologics treatment.

Feature	Anti-CENP-B-Negative			P	Anti-CENP-B-Positive			P
	M0 (median, IQR)	M6 (median, IQR)	M6-M0 (median, IQR)		M0 (median, IQR)	M6 (median, IQR)	M6-M0 (median, IQR)	
SLEDAI-2K	12 (10-12)	8 (6-10)	-3 (-4--2)	<0.001	12.5 (10.25-15)	6.5 (4.5-9.75)	-5 (-7.5--4)	0.001
24h-UTP(g/24h)	0.34 (0.07-2.98)	0.08 (0.02-0.78)	-0.09 (-1.73--0.02)	<0.001	0.74 (0.4-2.39)	0.1 (0.04-0.22)	-0.62 (-2.34--0.19)	0.008
dsDNA (IU/ml)	186.18 (54.54-300)	33.4 (10.79-107.38)	-93.32 (-199.09--9.33)	<0.001	192.65 (85.28-300)	14 (5.62-31.49)	-150.1 (-269.72--68.56)	0.001
CD16 + 56+NK(n/μL)	80 (46.25-124.75)	107.5 (68-189.75)	23.5 (-10.75-76.75)	<0.001	73.5 (58-91.25)	157 (96.22-216.25)	66.65 (6.98-108.25)	0.024
CD16 + 56+NK%	5.93 (3.81-9.83)	7.86 (4.26-11.43)	1.03 (-1.29-4.45)	<0.001	4.76 (3.12-7.93)	11.24 (7.36-12.84)	3.47 (1.96-7.52)	0.005
CD19+B(n/μL)	131 (73-257)	85 (43-181)	-22 (-113-19)	<0.001	259 (81.35-485)	86.5 (41-214)	-207.5 (-415.82-36.5)	0.069
CD19+B%	11.16 (6.02-18.3)	6.05 (3.11-10.72)	-2.53 (-8.9-0.14)	<0.001	16.45 (8.24-29.04)	6.71 (4.03-13.4)	-10.01 (-19.5-1.21)	0.028
CD3+CD4+T(n/μL)	486 (359.25-681.75)	578.5 (444-794)	73.5 (-73.75-223)	<0.001	491.5 (339.25-677.75)	516.5 (354.25-604)	-36 (-128.5-273)	0.754
CD3+CD4+T%	36.66 (29.61-42.33)	37.89 (30.88-45.08)	2.11 (-2.81-6.34)	<0.001	35.18 (25.52-44.18)	39.69 (36.5-43.28)	2.2 (-0.34-9.55)	0.209
CD3+CD8+T(n/μL)	483 (323-719.5)	573.5 (397.25-793.75)	76 (-98-213.75)	<0.001	500.5 (275.5-662)	476 (414.75-634.5)	101 (-87.75-197)	0.414
CD3+CD8+T%	37.37 (29.18-43.65)	37.88 (32.33-46.86)	0.59 (-3.47-5.7)	0.092	38.94 (23.91-41.65)	40.03 (36.32-43.02)	2.24 (-2.71-7.34)	0.346
CD3+T(n/μL)	1034 (742.75-1460.25)	1220.5 (952.75-1615.25)	153 (-138.5-507.75)	<0.001	988 (739.5-1338.5)	1041.5 (748.75-1339.75)	148 (-239.25-359.25)	0.490
CD3+T%	78.33 (69.85-83.83)	81.93 (76.11-86.02)	2.28 (-1.37-9.49)	<0.001	79.38 (64.78-84.5)	80.99 (75.91-85.32)	4.6 (-2.27-9.76)	0.187
IgA(g/L)	2.19 (1.53-2.96)	2.05 (1.32-2.56)	-0.16 (-0.6-0.19)	<0.001	2.68 (2.34-4.08)	2.04 (1.47-3.55)	-0.65 (-1.09--0.27)	0.060
IgG(g/L)	11.23 (8.34-14.31)	10.53 (7.73-12.95)	-0.45 (-4.28-1.06)	<0.001	17.38 (13.84-18.95)	12.68 (10.01-14.82)	-5.57 (-9.1--1.28)	0.012
IgM(g/L)	0.75 (0.41-1.22)	0.62 (0.45-1.02)	-0.05 (-0.29-0.12)	0.004	1.26 (0.8-1.96)	0.64 (0.43-0.96)	-0.62 (-1.19--0.07)	0.014
C3(g/L)	0.72 (0.55-0.88)	0.99 (0.81-1.15)	0.23 (0.06-0.45)	<0.001	0.62 (0.41-0.72)	0.97 (0.9-1.12)	0.44 (0.24-0.69)	0.001
C4(g/L)	0.14 (0.08-0.21)	0.19 (0.13-0.27)	0.05 (0-0.1)	<0.001	0.07 (0.06-0.13)	0.26 (0.21-0.33)	0.17 (0.05-0.26)	0.001

For the variables, the median values with interquartile range (IQR) were provided. SOC, standard of care; M0, baseline (pre-treatment); M6, six-month post-treatment. Paired Wilcoxon test was used for comparison and *p*<0.05 was considered statistically significant.

In the SOC + biologics (belimumab, telitacicept, or rituximab) arm (**Table 3**), the anti-CENP-B–negative group showed clear increase in T-cell subsets (CD3^+^, CD3^+^CD4^+^, CD3^+^CD8^+^) and CD3^-^CD16^+^CD56^+^ NK cells, together with a reduction in CD19^+^ B-cell numbers and percentages. In contrast, the anti-CENP-B–positive group exhibited only a significant decrease in CD19^+^ B-cell percentage (*p*=0.012), whereas both the percentage and absolute count of CD3^-^CD16^+^CD56^+^ NK cells increased markedly (*p*=0.024 and 0.005, respectively). Humoral indices (C3 and C4) increased in both groups; additionally, IgA, IgG and IgM fell significantly in the anti-CENP-B–negative group, while IgG and IgM declined noticeably in the anti-CENP-B–positive group.

Among the 73 anti-CENP-B–positive SLE patients, after a follow-up duration of six months, none of these individuals evolved into diffuse cutaneous SSc, limited cutaneous SSc, or fulfilled the 2013 ACR/EULAR classification criteria for SSc during the study period. Specifically, no patient developed calcinosis, Raynaud’s phenomenon not previously attributed to SLE, esophageal dysmotility, sclerodactyly, or telangiectasia (CREST spectrum).

Statistical analyses were performed to compare the relative reduction in SLEDAI-2K scores ((M0-M6)/M0) between anti-CENP-B–positive and –negative patients within each treatment arm (**Supplementary Figures S3A, B**). Although the decrease was similar for both serostatus groups receiving SOC alone, the reduction appeared more pronounced when SOC was combined with biologics. However, when treatment modalities were compared within each serostatus group (**Supplementary Figures S3C, D**), no statistically significant difference in SLEDAI-2K reduction was observed between SOC monotherapy and SOC plus biologics, regardless of anti-CENP-B status. Taken together, these findings indicate that, although anti-CENP-B–positive patients exhibited a numerically greater decline in disease activity with combination therapy, the observed immunological differences in NK/T cell frequencies did not translate into a statistically significant differential clinical response between the two serostatus groups.

## 4 Discussion

SLE is defined by disease-specific autoantibodies, among which ANA is the universal hallmark (25). Anti-Sm and anti-dsDNA are highly specific for SLE, whereas anti-Ro/SSA, anti-La/SSB, anti-U1RNP, anti-nucleosome, anti-histone, anti-Rib-P, anti-PCNA and anti-cardiolipin are less specific and may overlap with other autoimmune disorders (26). Anti-CENP-B, a marker typically linked to limited cutaneous systemic sclerosis (lcSSc), is rarely encountered in SLE; reported frequencies range from 2–11% (16, 17). In our regional cohort of 687 SLE patients, 73 (10.6%) were anti-CENP-B-positive, mirroring previous estimates. Whether this antibody shapes the clinical or immunological phenotype of SLE remains contentious; by mapping the full spectrum of features associated with anti-CENP-B positivity, our study furnishes a refined framework for understanding disease heterogeneity and for identifying patients who may benefit from tailored therapeutic strategies.

In this study, anti-CENP-B-positive SLE patients exhibited a distinct phenotypic profile characterized by significantly higher frequencies of Raynaud’s phenomenon, cardiac involvement (predominantly pericarditis), and pleuropulmonary manifestations (mainly pleuritis) relative to their anti-CENB-negative counterparts, whereas the incidence of LN was comparatively lower. Raynaud’s phenomenon is a well-recognized feature of systemic autoimmune diseases, documented in 30–40% of SLE, 15%–25% of primary Sjögren’s syndrome, and ≥ 95% of systemic sclerosis (SSc) cohorts (27). Importantly, none of our anti-CENP-B-positive SLE patients fulfilled criteria for CREST (calcinosis, Raynaud’s, esophageal dysmotility, sclerodactyly, telangiectasia) or developed other scleroderma-specific cutaneous or vascular changes, underscoring that Raynaud’s phenomenon, although shared, is insufficient to confer an SSc classification under current criteria (5, 28). Likewise, neither diffuse nor limited cutaneous SSc evolved in any of the 73 anti-CENP-B-positive individuals during follow-up, a finding consistent with prior reports (17). Collectively, these data indicate that anti-CENP-B positivity in SLE identifies a subset with heightened serosal and vasospastic features but does not presage progression to systemic sclerosis.

SLE presents with highly heterogeneous clinical and laboratory features, and a subset of autoantibodies has proven diagnostic and prognostic value (29). Here, anti-CENP-B-positive patients displayed a distinct serological signature: ANA, anti-dsDNA, AnuA and AHA titers were all significantly lower than in the anti-CENP-B-negative group. Anti-dsDNA, AnuA and AHA are prototypical SLE autoantibodies, each reported in 60–80% of patients (26); their relative paucity in the anti-CENP-B-positive cohort therefore indicates an atypical serological cluster. Both anti-dsDNA and AnuA are implicated in lupus nephritis (LN) through direct or indirect binding to renal antigens and correlate positively with SLEDAI-2K scores (30, 31). Consistent with these data, anti-CENP-B-negative patients exhibited more frequent LN, higher levels of these antibodies and greater disease activity. Collectively, the findings underscore marked serological heterogeneity within SLE and suggest that anti-CENP-B positivity identifies a subgroup with a less “classic” autoantibody repertoire.

Immunoglobulins, complement fractions, and lymphocyte subsets are consistently perturbed in SLE (32–34). We now show that anti-CENP-B–positive patients are distinguished by lower serum C4 and higher IgA, IgG, and IgM levels, together with an expanded CD19^+^ B-cell compartment and a contracted CD8^+^ T-cell pool. Although B-cell lymphopenia is typical of SLE, the accumulation of autoreactive clones is thought to drive progressive disease (34, 35); the parallel rise in immunoglobulins observed here supports this scenario in the anti-CENP-B–positive subset. T-cell dysregulation further amplifies autoimmunity and infection risk (36–38), and the inverse correlation between CD8^+^ T-cell and CD19^+^ B-cell frequencies across our cohort suggests a reciprocal relationship that may aggravate immune imbalance. Finally, the lower C4 values characteristic of anti-CENP-B positivity showed moderate negative correlations with 24h-UTP, SLEDAI-2K, anti-dsDNA titers, and IgG, implicating that C4 is excessively consumed or genetically determined deficiency——in both disease activity and susceptibility among these patients (39, 40). In anti-CENP-B-positive individuals, CD19^+^ B-cell abundance exhibited a weak-to-moderate inverse correlation with CD8^+^ T-cell percentage, indicating mild subset complementarity or a shift in immune balance between these two lymphocyte subsets. Reduced CD8^+^ T-cell levels impair the cytotoxic clearance of autoreactive B cells, thereby promoting their expansion and autoantibody production (41); this mechanism partly explains the elevated immunoglobulin (IgA, IgG, and IgM) levels observed in CENP-B-positive SLE patients.

SLE patients positive for anti-CENP-B differ markedly from seronegative cases in clinical phenotype, serology and immune profile. To dissect this heterogeneity, we first screened variables by univariate logistic regression and then entered them into a multivariate model; the resulting coefficients were used to build a nomogram that estimates the probability of anti-CENP-B positivity. Age, Raynaud’s phenomenon, CD19^+^ B-cell count and IgG level emerged as independent predictors. The association with older age agrees with previous data (15). Expanded CD19^+^ B-cell pools likely reflect autoreactive plasma-cell overgrowth, consistent with their documented role in autoimmunity and inflammation (35). Here, we show that IgA, IgG and IgM are simultaneously elevated in anti-CENP-B-positive SLE, implying heightened plasma-cell activity in these patients. The same synchronized hypergammaglobulinaemia has been described in anti-CENP-B-positive primary Sjögren’s syndrome, supporting the concept that this antibody marks a shared genetic endotype that spans systemic autoimmune diseases. Earlier pSS studies have reported variable immunoglobulin levels (42, 43); whether this discrepancy stems from ethnic background, concomitant therapy or analytical methods awaits clarification. Raynaud’s phenomenon, although common in the general population (5–15%) (44, 45), is an early hallmark of lcSSc, a disorder defined by anti-CENP-B reactivity (46). Its identification as an independent risk factor therefore mandates long-term surveillance for lcSSc development in anti-CENP-B-positive SLE patients.

A notable and seemingly paradoxical observation emerged from our analyses: the anti-CENP-B-positive cohort exhibited a significant expansion of CD19+ B cells alongside elevated levels of IgA, IgG, and IgM, yet concurrently displayed lower SLEDAI-2K scores, a reduced prevalence of severe organ involvement (LN), and decreased levels of pathogenic autoantibodies (anti-dsDNA, AnuA, AHA) compared to the anti-CENP-B-negative cohort. This finding stands in contrast to the conventional paradigm linking immune activation—characterized by B cell expansion and increased immunoglobulin production—to more severe disease activity in SLE. To reconcile this apparent contradiction, it is critical to revisit the complex and context-dependent roles of B cells in SLE pathogenesis. SLE is a canonical B cell-driven disease, yet the therapeutic efficacy of B cell depletion therapies (BCDTs) in SLE remains limited, even though BCDTs are effective in other autoimmune conditions such as rheumatoid arthritis and multiple sclerosis. This discrepancy is likely attributable to the heterogeneity, specificity, and complexity of SLE (47, 48). A key mechanistic distinction lies in the subset of B cells driving pathology: in SLE, the extrafollicular generation of class-switched B cells, rather than the predominantly non-class-switched IgM^+^ B cells residing in B cell follicles or germinal centers, plays the pivotal pathogenic role (47, 49). Beyond this subset-specific effect, the simplistic view that plasma cells and antibodies are uniformly “pathogenic” has evolved into a more nuanced understanding that B cell functions are highly context-dependent, encompassing both pathogenic and protective roles (47). Thus, the seemingly paradoxical findings in the anti-CENP-B-positive cohort may be explained by adopting a holistic perspective on the microenvironment in which anti-CENP-B antibodies are generated, where the expanded B cell population and elevated immunoglobulins may reflect a protective, rather than pathogenic, B cell response.

Treatment patterns differed sharply between anti-CENP-B–positive and –negative patients: 80.8% (59/73) received standard-of-care (SOC) alone, and only 19.2% (14/73) were escalated to SOC plus biologics (belimumab, telitacicept, or rituximab) in accordance with the 2020 Chinese SLE guidelines (22). In anti-CENP-B–positive SLE patients, intensification with SOC plus biologics (belimumab, telitacicept or rituximab) conferred no additional benefit. Both regimens demonstrated similar changes from baseline: CD3^-^CD16^+^CD56^+^ NK-cell numbers and percentages increased, CD19^+^ B-cell percentages decreased, yet no T-cell subset improved significantly; In the SOC-alone arm, the CENP-B-positive subgroup showed a pronounced rebound in T-cell counts, a decrease in IgG and IgM, and an increase in C3 and C4 after treatment; SLEDAI-2K scores, anti-dsDNA titers, and 24h-UTP all declined. These indices changed concordantly across the treatment groups, indicating that the SOC + Biologics regimen conferred no additional benefit to CENP-B-positive patients. These results accord with previous reports that SOC plus biologics adds no measurable value in a defined subset of SLE patients (50–52).

The limited therapeutic benefit of biologics in anti-CENP-B-positive SLE patients may be attributed to the unique immunological and genetic characteristics of this subgroup, which aligns with the marked heterogeneity of SLE and the targeted nature of biologic therapies. A key contributing factor could be the activation of alternative inflammatory pathways not effectively targeted by current biologics: for instance, microRNA-152-3p-regulated DNMT1/MyD88 signaling, which modulates Toll-like receptor (TLR)-mediated inflammation critical to SLE pathogenesis (53), may drive persistent immune activation independent of the cytokine or immune pathways targeted by most biologics. Additionally, the distinct functional state of NK cells and their interactions with other immune components in anti-CENP-B-positive patients could influence treatment efficacy, as existing biologics may not adequately modulate NK cell activity linked to immune regulation and chronic inflammation (54). Furthermore, specific genetic markers such as HLA-DRB1 alleles, which have been associated with variable SLE disease severity and treatment responses (55), may predispose this subgroup to an immunological profile—including a distinct cytokine milieu or immune cell activation pattern—that is not addressed by biologics designed for the broader SLE population. Collectively, these unique features of anti-CENP-B-positive SLE patients may explain the observed differential response to biologic therapies.

In contrast, anti-CENP-B–negative patients treated with SOC plus biologics (belimumab, telitacicept or rituximab) derived a clear therapeutic advantage: all T-cell subsets and CD3^-^CD16^+^CD56^+^ NK cells increased markedly, whereas CD19^+^ B cells declined. Humoral immunity improved, as evidenced by falls in IgA and IgG and restoration of C3 and C4. Disease-activity indices (SLEDAI-2K scores, anti-dsDNA titers and 24h-UTP) decreased significantly, producing substantially greater benefit than SOC alone. These findings probably reflect the high prevalence of LN in this group (48.5%, 115/237), because published data show that SOC plus biologics confer definite renal protection in LN (35). Collectively, the present findings suggest that SLE patients positive for anti-CENP-B antibodies may constitute a distinct subtype of SLE.

Although the study delineates a distinct subtype of SLE defined by anti-CENP-B positivity and integrates clinical, biochemical, immunologic and therapeutic-outcome data, several limitations merit consideration. First, the number of anti-CENP-B–positive patients was modest and the cohort was confined to central China; we are therefore establishing a multi-province consortium to validate the generalizability of our findings. Second, the follow-up interval was limited to six months; longer-term observation is required to determine the durability of treatment responses and the propensity of these patients to evolve systemic sclerosis, primary Sjögren’s syndrome, primary biliary cholangitis or other overlapping autoimmune diseases. Such studies will refine the characterization of the anti-CENP-B–positive SLE subtype and facilitate more precise clinical management.

## 5 Conclusion

In this study, our results indicate that anti-CENP-B positivity may delineate a distinct SLE subset characterized by unique clinical and serological features, warranting individualized treatment plans and judicious use of biologic agents. Ongoing surveillance for potential progression to systemic sclerosis is also advised, given the well-documented serologic overlap between these conditions.

## Data Availability

The original contributions presented in the study are included in the article/**Supplementary Material**. Further inquiries can be directed to the corresponding authors.
