# Supplementary material for: Anti-CENP-B polarity divides SLE: divergent clinical-immune phenotypes and distinct treatment responses
Source: Front Immunol. 2026 Jan 22;17:1762899. doi: 10.3389/fimmu.2026.1762899 (PMC12872543; doi:10.3389/fimmu.2026.1762899)
Supplement: Supplementary file 2 [file DataSheet1.pdf]

Supplementary files

Part 1. Definitions of the organ involvements.

These definitions were derived from the SLICC-2012 and EULAR/ACR-2019 criteria and corroborated by objective investigations, as follows:

Cardiac: a) Acute pericarditis: ECG ST-elevation or new pericardial effusion on echocardiography, plus pleuritic chest pain or friction rub. b) Myocarditis: left-ventricular dysfunction on echocardiography or cardiac MRI not explained by ischemia, accompanied by elevated troponin. c) Libman-Sacks endocarditis: vegetations documented by echocardiography.

Pleuropulmonary: a) Pleuritis: pleuritic pain plus audible rub or imaging-demonstrated effusion/thickening. b) Lupus pneumonitis: new pulmonary infiltrate with infection excluded (negative cultures/PCR) and rapid response to immunosuppression. c) Pulmonary hemorrhage: hemoptysis, alveolar shadowing on imaging, and ≥20 % hemosiderin-laden macrophages in bronchoalveolar lavage. d) Interstitial lung disease: HRCT showing reticular or ground-glass opacities plus DLco <70 % predicted.

Musculoskeletal: a) Arthritis: swelling or tenderness in ≥2 joints with synovitis confirmed by ultrasound or MRI. b) Myositis: proximal muscle weakness, elevated creatine kinase or aldolase, compatible EMG or MRI findings, or biopsy evidence of perivascular/endomysial inflammation.

Renal: Proteinuria ≥500 mg per 24 h or urine albumin-to-creatinine ratio ≥0.5 g/g together with active urinary sediment (>5 red blood cells per high-power field or red-cell casts), or histological class per ISN/RPS 2003 on renal biopsy.

Hematologic: a) Hemolytic anemia: increased reticulocytes, decreased haptoglobin, and positive direct or indirect Coombs test. b) Leucopenia: < 4 000/μL on at least two separate occasions. c) Thrombocytopenia: <100 000/μL on at least two separate occasions, or biopsy-proven immune thrombocytopenia.

Neuropsychiatric: a) Seizure: diagnosis confirmed by a neurologist with metabolic and drug causes excluded. b) Psychosis: fulfilling DSM-5 criteria with steroid-induced psychosis excluded. c) Cerebrovascular disease: imaging-proven infarct or hemorrhage satisfying ACR neuro-SLE definitions. d) Peripheral neuropathy: EMG confirmation with diabetes, drugs, and other causes excluded.

Gastrointestinal: a) Lupus enteritis: abdominal pain accompanied by CT "target sign" or bowel-wall thickening >3 mm in the absence of infection. b) Protein-losing enteropathy: serum albumin <30 g/L plus elevated fecal α-1-antitrypsin clearance. c) Peritonitis: ascitic polymorphonuclear cell count >250/μL with negative bacterial culture.

Part 2. Follow-up of the patients for SSc occurrence:

According to the 2013 ACR/EULAR classification criteria and the CREST syndrome constellation: (1) Skin: No patients exhibited skin thickening, digital sclerosis, fingertip ulcers, or pitted scarring; (2) Vascular: 15 patients had Raynaud's phenomenon at baseline, 10 of whom achieved remission after therapy; nail-fold capillaroscopy was not routine, but no digital pits, ulcers, or visible capillary abnormalities were found on annual nail-fold inspection conducted within the 6-month follow-up; (3) Pulmonary: All patients underwent at least one HRCT and echocardiogram during the 6-month follow-up, with no new interstitial lung disease or pulmonary arterial hypertension (tricuspid regurgitation velocity, TRV > 3.4m/s, Additional echocardiographic signs supportive of pulmonary hypertension (PH)) was detected) detected; (4) Renal: No cases of scleroderma renal crisis were identified during follow-up; (5) Serology: Of the 73 patients who were anti-CENP-B-positive at baseline, 6 seroconverted to anti-CENP-B-negative and 2 anti-Scl-70-positive patients seroconverted to negative within the 6-month follow-up period, making early SSc unlikely; (6) CREST: No patients met ≥2 criteria for calcinosis, Raynaud's phenomenon, esophageal dysmotility, sclerodactyly, or telangiectasia during follow-up. In summary, among these 73 patients, none accrued ≥9 points on the 2013 ACR/EULAR criteria for definite systemic sclerosis after 6 months of follow-up.

Anti-CENP-B-positive cases (n = 73)		
SSc-specific features	M0 (n = 73)	M6 (n = 73)
Skin thickening of the fingers of both hands extending proximal to the metacarpophalangeal joints (sufficient criterion)	0	0
Skin thickening of the fingers	0	0
Fingertip lesions	0	0
Telangiectasia	0	0
Abnormal nailfold capillaries	-	-
Pulmonary arterial hypertension and/or interstitial lung disease	0	0
Raynaud's phenomenon	15	5
SSc-related autoantibodies: anti-centromere	73(+)	67(+)
anti–Scl-70	2	0
anti–RNApolymeraseIII	0	0

Part 3. Supplementary figures.

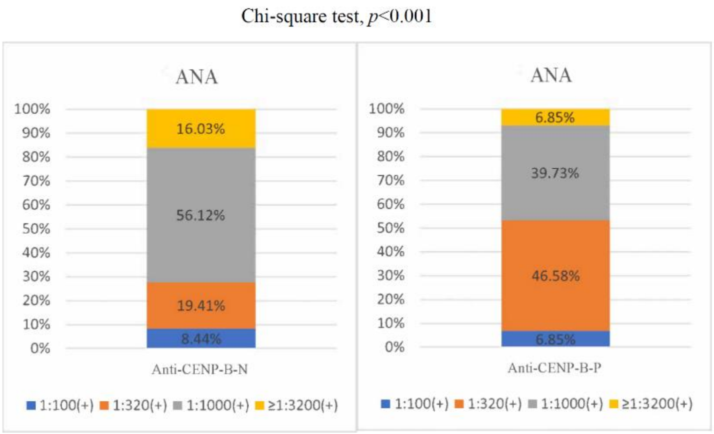

Figure S1. Comparison of ANA titers between anti-CENP-B-N and anti-CENP-B-P groups. Chi-square test was used for comparisons and $p < 0.05$ was considered significant. The titers of 1:100, 1:320, 1:1000, and ≥1:3200 indicated low, moderate, strong, and very strong ANA levels in the serum, respectively.

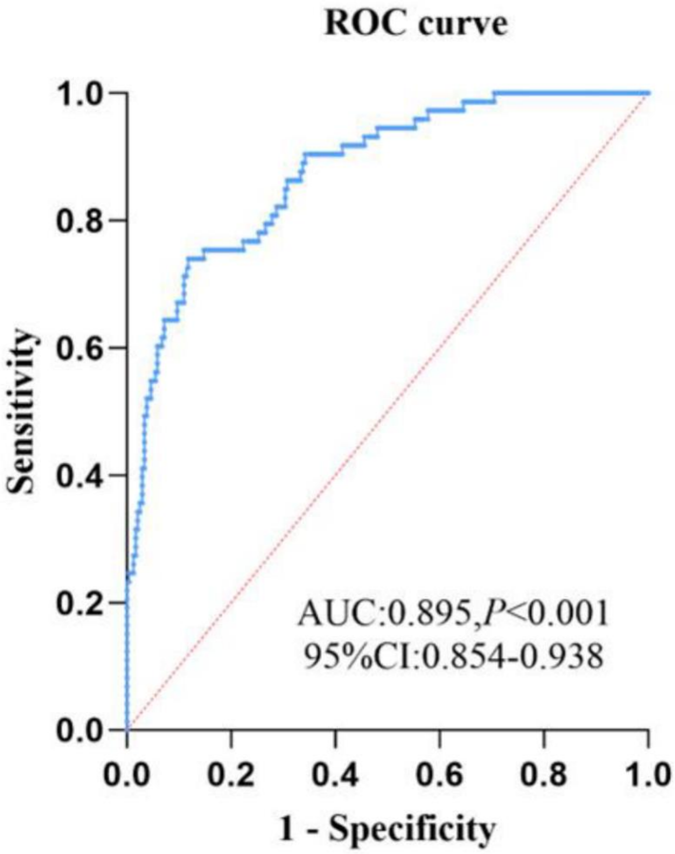

Figure S2. ROC curve of the multivariate model in discriminating anti-CENP-B status in SLE patients.

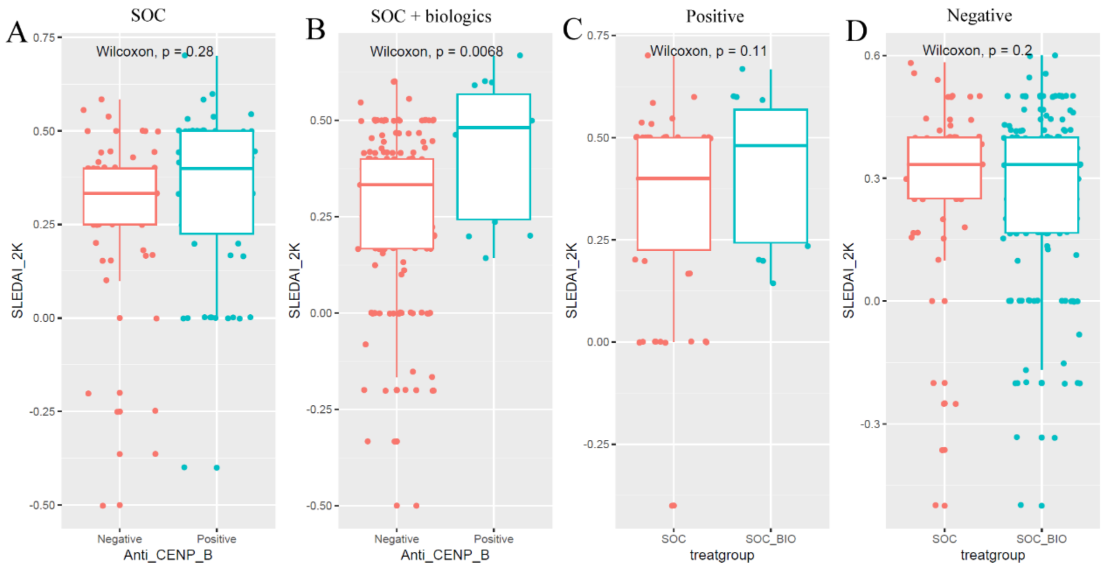

Figure S3. The SLEDAI_2K decrease in different patients due to anti-CENP-B status or different treatments. (A-B) The comparisons of SLEDAI_2K decrease in anti-CENP-B positive and negative SLE patients between and after the same treatment. (C-D) The comparisons of SLEDAI_2K decrease in anti-CENP-B positive or negative SLE patients due to different treatments. Paired Wilcoxon test was used and $p < 0.05$ was considered significant.
